# Genome sequences of *Aspergillus fumigatus* strains isolated from wildfowl in Southern Ontario, Canada

**DOI:** 10.1128/mra.00294-25

**Published:** 2025-10-20

**Authors:** Oscar Romero, Magalie Galarneau, Samanta Pladwig, Boyan Liu, Sherri Cox, Jennifer Geddes-McAlister

**Affiliations:** 1 Molecular and Cellular Biology Department, University of Guelph 3653, Guelph, Ontario, Canada; 2 Department of Integrative Biology, University of Guelph 177372, Guelph, Ontario, Canada; University of California Riverside, Riverside, California, USA

**Keywords:** *Aspergillus*, fungi, genome, antifungal resistance

## Abstract

We present the genome sequences of four *Aspergillus fumigatus* strains isolated from infected wildfowl collected from Wildlife Rehabilitation Centers in Southern Ontario, Canada. Phenotypic and phylogenetic assessment confirmed infection with *A. fumigatus,* suggesting a correlation between fungal infection and wildfowl mortality.

## ANNOUNCEMENT

Invasive aspergillosis (IA) is a fungal infection caused by *Aspergillus* species affecting humans and animals, including birds (1). Such infections have severe impacts on host health, and rising rates of antifungal resistance limit the efficacy of current treatments (2). In this study, we isolated *Aspergillus fumigatus* from four wildfowl submitted to wildlife rehabilitation centers in Southern Ontario, Canada, with suspected cases of IA (3). Specimens included a male osprey (isolate: AfB2), a female peregrine falcon (isolate: AfB6), and male (isolate: AfB7) and female (isolate: AfB8) broad-winged hawks.

**Fig 1:**
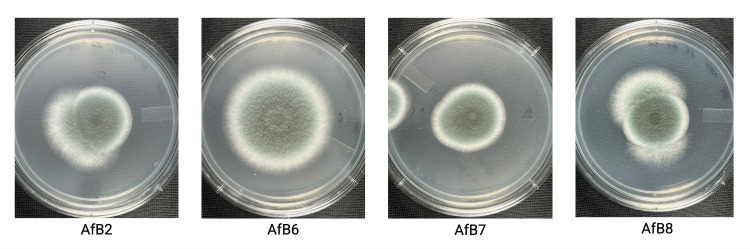
Solid cultures of *A. fumigatus* strains isolated from wildfowl in Southern Ontario, Canada, following 3 d of growth on PDA at 37 °C.

Within the avian respiratory tract (e.g., lungs and air sacs), tissue samples were collected from aspergillosis-like lesions. Samples were homogenized in microcentrifuge tubes containing 0.8 g of steel beads (2 mm, Fisher Scientific) and 1 mL of phosphate-buffered saline (PBS) using a bullet blender (Next Advance) for 2×5 min cycles. Visual assessment of fungal growth and *Aspergillus* spp. identification was performed by plating serial dilutions (10^2^, 10^1^) of homogenates on yeast peptone dextrose ( 1% yeast extract, 2% peptone, 2% glucose, 1.5% agar) plates for 7 d at 30 °C. The cultures yielded green mold-like colonies, characteristic of *Aspergillus* spp. (Fig. 1) (4). Next, a swab of the initial culture was plated for 5 d at 37 °C on potato dextrose agar (PDA; 20% potato infusion, 2% glucose, 1.5% agar). Following sufficient growth, a conidial resuspension was prepared using 5 mL PBS-Tween-20 (Thermo Scientific). Genomic DNA was extracted from the conidial suspension with the Fungi/Yeast Genomic DNA Isolation Kit (Norgen Biotek) as per manufacturer instructions. Illumina sequencing libraries were prepared with the Illumina DNA Prep Kit and custom IDT 10 bp unique dual indices with a target insert size of 280 bp. Sequencing was performed on an Illumina NovaSeq X Plus sequencer in one or more multiplexed shared-flow-cell runs, producing 2×151 bp paired-end reads. Default parameters were applied for bioinformatic tools, unless otherwise specified. Demultiplexing, quality control, and adapter trimming were performed with bcl-convert v4.2.4 (5). The short read assembly was performed with Unicycler v0.5.0 (6), and the analysis was recorded with Quast v5.2.0 (Table 1) (7). The genomes were annotated with Funannotate v1.8.15 (8) with InterProScan and eggnog mapper enabled.

**TABLE 1:** Genome assembly statistics for four *A. fumigatus* isolates collected from wildfowl in southern Ontario, Canada

Assembly	# contigs	Largest contig	Total length	GC (%)	N50	Coverage	Completeness
AfB2	279	760,125	28,135,400	49.47	221,218	152.68 ×	97%
AfB6	255	1,001,284	27,990,059	49.54	264,710	148.03 ×	97.7%
AfB7	293	703,397	27,958,471	49.46	214,179	168.96 ×	97%
AfB8	294	787,527	28,065,029	49.46	210,363	178.97 ×	96.8%

## Data Availability

The genome sequences of these A. fumigatus strains are publicly available in GenBank under the accession numbers: JBJNTG000000000 (AfB2), JBMETL000000000 (AfB6), JBJNTF000000000 (AfB7), and JBJNTE000000000 (AfB8). The raw reads were deposited in the Sequence Read Archive (SRA) under the accession numbers SRX28067732 (AfB2), SRX28067733 (AfB6), SRX28067734 (AfB7), and SRX28067735 (AfB8).
